# DIMENSIONS OF SOCIAL SUPPORT AND ASSOCIATIONS WITH HEALTH AMONG OLDER ADULTS

**DOI:** 10.1093/geroni/igz038.1165

**Published:** 2019-11-08

**Authors:** Samuel Asante, Eun-Jun Bang

**Affiliations:** Northeastern State University, Broken Arrow, Oklahoma, United States

## Abstract

Social support is fundamental to human survival, and is significantly involved in the attainment and maintenance of good health and wellbeing. Previous studies have often considered social support as a singular, non-dimensional construct. While this is important and enlightening, the method of adding up individual aspects to create a singular, non-dimensional construct has produced little understanding of these aspects/dimensions of social support and their implications for health. This study examined three dimensions or types of social support-affective, confidant, and instrumental support-and their associations with physical and mental health in older adults. Data for this study were obtain from Utah Fertility, Longevity, and Aging (FLAG) study. Participants involved 325 older adults, aged 50 years or older. Results showed a significant, strong positive correlation between affective support and physical and mental health, and weak association between confidant support and physical and mental health. The correlation between instrumental support and physical and mental health was moderate. After controlling for the influence of socio-demographic variables, affective and instrumental support significantly predicted physical and mental health. Confidant support was not a significant predictor of either physical or mental health. The findings suggest both affective and instrumental support might be relatively more important to the health and mental wellbeing of older adults than confidant support, underscoring the relative importance older adults attach to quality rather than confidant support, which essentially is quantity of social ties.

